# 

**Published:** 1879-12

**Authors:** 


### CONTENTS.

### COMMUNICATIONS.

The New and the Old and the New Departure by an Old One. *By J. A. Robinson, D. D. S.* ........ 493
Historical Sketch of the Discovery of the Composition of Water. *By E. G. Betty, D. D. S.* ........ 506
Dental Students. *By D. C. Hawxhurst* ........ 514
Dr. Crawford W. Long, the Discoverer of Anæsthesia. Anæsthesia and Anæsthetics ........ 518
Trophic Neuralgias. *By John J. Caldwell, Baltimore, Md* ........ 521

### SELECTIONS.

How Far Can We Hear with the Telephone? ........ 522
Air as a Stimulant ........ 523
India Rubber ........ 524
Statistics of Age ........ 524
Scientific News ........ 525

### EDITORIAL.

The Gardner Case—Correction ........ 528
Bibliographical ........ 532

